# Accuracy of the Double Variation Technique of Refractive Index Measurement

**DOI:** 10.6028/jres.097.033

**Published:** 1992

**Authors:** Jennifer R. Verkouteren, Eric B. Steel, Eric S. Windsor, John M. Phelps

**Affiliations:** National Institute of Standards and Technology, Gaithersburg, MD 20899

**Keywords:** dispersion, double variation, immersion method, optical properties, polarized light microscopy, refractive index

## Abstract

Errors in the double variation teclinique of refractive index measurement are analyzed using a new approach. The ability to measure matching wavelength is characterized, along with the effect on the calculated refractive index. Refractive index accuracy and precision are very dependent on the specifics of each calibration set, particularly the difference in dispersion between the liquid and solid. Our best precision (±1 or 2×10^−4^) is attained only when the difference in dispersion between liquid and solid is small, and is dependent on an individual operator’s ability to perceive changes in relief. This precision is impossible to achieve for the other glass/liquid combinations, where we are limited by a precision of approximately 1 nm in the selection of matching wavelength. A bias in the measurement of matching wavelength exists that affects the accuracy of the calculated refractive indices. The magnitude of the bias appears to be controlled by the bandpass of the graded interference filter. The errors in refractive index using a graded interference filter with a bandpass of 30 nm FWHM (full width at half maximum intensity) are an order of magnitude larger than the errors using a filter with a bandpass of 15 nm FWHM.

## 1. Introduction

One of the tasks in our laboratory was to characterize the optical properties of asbestos minerals to serve as NIST Standard Reference Materials (SRMs). Mine-grade chrysotile, amosite, and crocidolite were to be made available as SRM 1866 [1] with certified values for all optical properties, and in particular, refractive index. The SRM is intended to serve as a calibration standard for laboratories that analyze bulk materials for asbestos using polarized light microscopy. Refractive index is a primary optical property used to characterize transparent minerals, including asbestos, during microscopic analysis. Immersion techniques for microscopic measurement of refractive index such as Becke line, oblique illumination, and focal masking are used routinely for refractive index measurement to the third place [2]. To characterize reference materials for use with these techniques, we need to use a method with higher accuracy and precision.

There are several microscopic techniques to measure refractive index accurately to the fourth place, including interferometry [3], the variation of temperature at constant wavelength (single variation) [4], and the double variation technique [5–8]. We decided to use the double variation technique for our measurements because we expected it to be an improvement on the single variation technique. The double variation technique requires the control of temperature and wavelength to match the refractive index of the liquid to that of the unknown solid. The solid is immersed in a liquid of known refractive index and dispersion, the temperature is held constant at some value between 20 and 35 °C, and the wavelength of the illuminating light is varied until the refractive index of the solid and liquid are observed to match, as indicated by a minimum of contrast. The temperature is then set to a new value and the process of determining the wavelength at which solid and liquid match in refractive index is repeated. The refractive index of the solid is calculated for each set of temperature/matching wavelength measurements using the dispersion equation and temperature coefficient of the liquid. The refractive indices thus determined are fit to standard dispersion equations, such as the Cauchy or Sellmeier equation [9] to describe the dispersion of the solid throughout the measured range of wavelengths.

References [6,7] describe a bias in refractive index measurements with respect to wavelength using the double variation technique which the authors attribute to the color sensitivity of the human eye. The measured values are biased high at wavelengths < 555 nm and are biased low at wavelengths >555 nm. The systematic errors reported are on the order of ±2×l0^−4^ Reference [8] describes an approach to correcting the bias by measuring a glass calibration standard closely matched in refractive index to the unknown. A refractive index correction value for each wavelength (Δ*n_λ_*) is calculated as *n*_meas_−*n*_true_ for the glass in question and is then applied to the measurements of the unknown, resulting in an accuracy and precision of approximately ± 1 × l0^−4^.

We obtained calibration glasses with refractive indices closely matched to the asbestos minerals and placed the appropriate glass in the heating stage alongside the asbestos mineral as an internal standard. We observed a bias with respect to wavelength of the same general nature reported earlier, however, the magnitude of the bias was at least a factor of ten larger. The errors were too large to correct for in the manner described in Ref. [8]. We began a systematic study of the variables involved in the measurement process and developed a different approach to assessing the errors in refractive index. This approach, in which we characterize the errors in the determination of the wavelength at which solid and liquid match in refractive index at each temperature, as opposed to characterizing the errors in refractive index with wavelength, provides a better understanding of the variables which control the accuracy and precision of the technique. This error analysis also allowed us to determine information concerning the measurement bias and the variables that control it.

## 2. Calibration

The calibration procedures for the equipment used in the measurement process and the results of the calibrations are given below. For a more detailed description of the types of equipment used in the double variation technique, including heating stages, illumination sources, immersion liquids, and refractometers, see Ref. [6].

### 2.1 Filter Calibration

We used three different filters in conjunction with the quartz halogen light source on our microscope: 1) a narrow-bandpass graded interference filter (GIF), 2) a broad-bandpass GIF, and 3) a set of seven fixed-wavelength interference filters. The narrow-bandpass GIF has a minimum bandpass of 15 nm full width at half the maximum intensity (FWHM), the broad-bandpass GIF has a minimum bandpass of 30 nm FWHM, and the fixed-wavelength filters each have a bandpass of 10 nm FWHM. A GIF is a 20 cm long rectangular interference filter that grades in thickness from one end to the other, allowing the selection of peak wavelengths from approximately 350 to 750 nm. The filter is marked from 1 to 200 in millimeter increments, and is calibrated at 16 positions by the manufacturer to determine the correspondence between filter position and peak wavelength. The filter holder has an exit slit which can be opened to a maximum width of 20 mm. The bandpass of the transmission peak is increased by opening the exit slit. The set of seven fbced-wavelength filters is designed to isolate the common spectral lines in the visible spectrum with transmittance peaks at 405, 436, 486, 546, 577, 589, and 656 nm.

The filters were each calibrated for wavelength and bandpass using a spectrophotometer. The spectrophotometer was first calibrated for wavelength at 589.3 (the mean of the doublet at 589.6 and 589.0 nm) using a sodium arc lamp, and at 546.1 and 435.8 nm using a mercury arc lamp. The filters were then calibrated using a tungsten light source to transmit white light through the filter, collecting the transmitted light with the spectrophotometer. The data were corrected for both the source characteristics and the relative sensitivity of the detector using a blank spectrum collected under the same conditions. The peak wavelength of the transmission curve is determined as the centroid of the peak, and the bandpass is measured graphically. The GIFs were calibrated at eight positions of the filter, corresponding to a range in wavelength of 440–630 nm, with the exit slit fixed at 2 mm. The narrow-bandpass GIF was also measured at one position (wavelength) but with a variation in slit width of 2–20 mm.

Our wavelength measurements for one of the GIFs disagreed with the manufacturer’s calibration measurements by 8 nm, with the same 8 nm discrepancy at each measured position. The measurements of the other GIF and the fixed-wavelength filters agreed with the manufacturers’ values. The bandpasses at FWHM for the two GIFs determined with the exit slit at 2 mm agree with the manufacturer’s values. (The manufacturers do not specify a slit size for their bandpass measurements.) If the exit slit is much narrower than 2 mm, the amount of light transmitted is insufficient to illuminate the microscope field. The transmission of a filter that is graded in thickness is complex, and the changes due to changing the size of the exit slit are difficult to predict. We measured the transmission of the narrow-bandpass GIF as a function of slit size, opening the slit symmetrically about the center position. The FWHM of the transmission peak increased from 15 nm for a 2 mm slit, to 32 nm at the fully open position (20 mm). The shape of the transmission curve also changed with slit size, with the FWHM increasing at a greater rate than the width at 1% of the peak height. Therefore, although the FWHM of the two GIFs are both approximately equivalent when the exit slit of the narrow-bandpass GIF is fully opened and the exit slit of the broad-bandpass GIF is at the minimum operating width, the transmission peaks are not equivalent.

The two GIFs differ in bandpass (15 or 30 nm FWHM) and also in transmission efficiency. The manufacturer states a transmission efficiency of 60% for the narrow-bandpass GIF and 30% for the broad-bandpass GIF. We did not measure the transmission efficiency of the filters; however, the change in brightness of the field of view supports the relative difference in efficiency given for the two GIFs. We could also see a difference in field illumination using the two GIFs that may reflect the difference in bandpass. Using the broad-bandpass GIF, we could see faint Becke lines with different colors than the field, such as red and blue Becke lines on a green field, whereas with the narrow-bandpass GIF and the fixed-wavelength filters, the Becke lines are simply intensity variations of the field color. The transmission efficiency of each fixed-wavelength filter is listed as approximately 60%, which again is in relative agreement with our observations of brightness in the field of view.

### 2.2 Immersion Liquids

The immersion liquids used in this study were obtained from Cargille.^1^ The refractive index at 589.3 nm was measured on a Zeiss Abbe-type refractometer (with an Amici prism) and a Bellingham and Stanley precision refractometer (without an Amici prism). The temperature was measured on the Zeiss refractometer with the instrument’s fixed thermometer, and the temperature on the Bellingham and Stanley refractometer was measured with an E-type thermocouple placed on the measuring prism. The refractive index measurements of the liquids are within ±2×10^−4^ of the value given by Cargille. In addition, the value *n*_F_−*n*_C_, which is a measure of the dispersion between 486.1 nm (F) and 656.3 nm (C), was determined for each liquid on the Zeiss refractometer using the Amici prism, and was again found to be within ±2× 10^−4^ of the value given by Cargille. Therefore, the dispersion equations of the liquids (Cauchy equations) provided by Cargille were used in this study.

We found that the errors in measurements at wavelengths other than 589.3 using the Bellingham and Stanley refractometer precluded the use of the data to determine the dispersion of the liquids independently. The errors were determined by measuring three SRMs designed for testing refractometers; SRM 1822 [10] and SRM 1823 [11]. The question of uncertainty in the liquid dispersions is addressed later in this paper.

### 2.3 Calibration Glasses

The glasses used in this study were obtained from D. Blackburn and D. Kauffman of the Mechanical Properties Group at NIST. The glasses were made at NIST approximately 30 years ago, and the refractive indices were characterized at that time using the minimum deviation technique [9] which is a high accuracy technique commonly used for the measurement of glasses. The technique requires that the glass be in the form of a prism with polished surfaces. The nomenclature used to identify the glasses when they were prepared was retained in this study. Each of the four glasses is identified by an alphabetic character followed by three or four digits. The refractive indices of some of the glasses were measured prior to this study by M. Dodge at NIST using the minimum deviation technique. The results agree with the earlier measurements and are accurate to ±5×10^−5^. The values at four wavelengths distributed across the visible spectrum were fit to a Cauchy equation using a least squares fit. A fit to a linearized Sellmeier equation [7] did not differ from a fit to a Cauchy equation by more than ±5 × 10^−6^, which is within the level of accuracy of the data; thus the mathematically simpler Cauchy equation and fit were used.

### 2.4 Heating Stage

The performance of the thermocouple in the heating stage during a temperature ramp was determined by placing a reference thermocouple in the immersion cell with its tip in close proximity to the stage thermocouple. We detected a problem in one of our commercial stages using this procedure. We found that the temperature readings were normal at room temperature and became anomalously high with increasing temperature. The thermocouple was not out of calibration, but instead was receiving additional heat from the wire used to heat the metal stage, and therefore was not providing a reliable measurement of the temperature of the immersion liquid. This problem was rectified by changing the placement of the stage thermocouple to remove it from proximity to the heating wire. A new heating stage was constructed in-house with this new design.

The thermocouple in the heating stage is accurate to ±0.1 °C at all temperatures between room temperature and 35 °C, and there is no temperature gradient in the immersion cell. The compositional stability of the liquids with temperature was tested by cycling the temperature up and down while measuring the refractive index of the calibration glasses. The liquids were found to be stable through at least two repeated ramps.

## 3. Experimental Design

We tested the measurement accuracy of the double variation technique using glasses with well characterized refractive indices that cover the range of the asbestos minerals. There are seven sets of glass/liquid calibration data, with a set defined as the measurements of one glass in one liquid. The liquids and calibration glasses are listed in Table 1 along with the wavelength range over which the calibration measurements were performed.

Refractive index is measured by placing grains of the appropriate glass in the calibrated liquid to select the wavelength at which solid and liquid match at the given temperature. A match is indicated by a minimum of relief. At matching conditions, the dispersion curve of the glass [Eq. (1)] and the dispersion curve of the liquid [Eq. (2)] intersect. Cauchy equations, as given in Eqs. (1) and (2), are commonly used to describe the dispersion of materials in the visible region of the spectrum [9].

$$n_{\text{gls}}=a+\frac{b}{\lambda^{2}}+\frac{c}{\lambda^{4}},$$ (1)

$$n_{\text{liq}}=d+\frac{e}{\lambda^{2}}+\frac{f}{\lambda^{4}}+\left[\left(T-25\right)\cdot dn/dT\right].$$ (2)

Temperature coefficients (d*n*/d*T*) of the liquids are negative, and are on the order of 5 × 10^−4^/°C. Temperature coefficients of most glasses are two orders of magnitude lower and are usually neglected for this range of temperature. The effect of glass temperature coefficients is discussed later in the paper.

For any given intersection (m) of the two dispersion curves, refractive index *(n)*, wavelength (*λ*), and temperature *(T)* are uniquely defined. To determine the accuracy and precision of our measurements, we need to know the set of (*n*. *λ*, *T)_m_* for *T* = 20–35 °C, which is our temperature range, for each calibration set. All possible values of *n*_m_ are given by the dispersion equation of the glass. Setting Eqs. (1) and (2) equal to each other and rewriting for *T* allows *T*_m_ to be calculated, as given in Eq. (3):

$$\begin{array}{l} \text{for}\;n_{\text{gls}}=n_{\text{liq}}:\\ T_{m}=\frac{a+\frac{b}{\lambda^{2}}+\frac{c}{\lambda^{4}}+\left(25\cdot dn/dT\right)-\left(d+\frac{e}{\lambda^{2}}+\frac{f}{\lambda^{4}}\right)}{dn\;dT}. \end{array}$$ (3)

The resulting (*T*, *λ*)_m_ data calculated from Eq. (3) for each calibration set were fit by least squares to a polynomial using DATAPLOT [12] to generate an expression that solves for *λ*_m_ at any *T*_m_. For all calibration sets, a quadratic or cubic equation was sufficient to achieve residuals from the fit of *λ*_m_ to *T*_m_ of ≤ 0.05 nm.

Because of the relationship among the variables *n*, *λ*, and *T*, we can look at our measurement errors in terms of any of the three. We chose to analyze the errors in *T* and *λ*, since they are the variables that we measure directly, and apply the results to the calculation of *n*. Due to the design of the experiment, in which we hold temperature constant during the measurement and vary wavelength to make a match, it is appropriate to view temperature as the independent variable and wavelength as the dependent variable, and determine our errors in measuring *λ*_m_. This assignment of variables fits the assumptions of most statistical approaches in which there is little or no error in the independent variable, and all error in the dependent variable. The same assumption of error does not hold however, if the data are analyzed in the more conventional way as (*λ*, *n*) pairs.

**Fig. 1:**
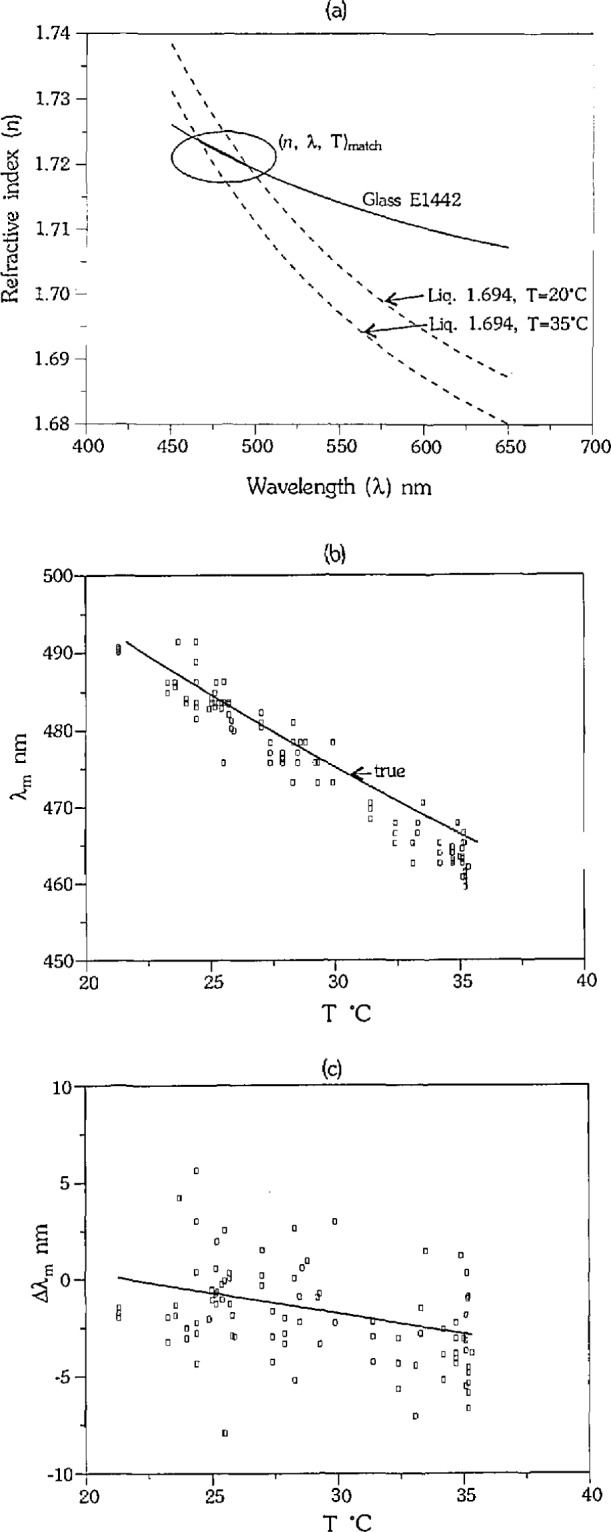
Generation of Δ*λ*_m_: (a) segment of E1442 dispersion curve measured in liquid 1.694 between 20 and 35 °C given by bold line, (b) calibration measurements (squares) vs true (solid line), and (c) errors calculated as *λ*^meas^ − *λ*^true^ (squares) with least squares linear fit (solid line).

Figure 1a illustrates the double variation technique and the relationship of *n*, *λ*, and *T* in the measurement of calibration glass E1442 in liquid 1.694 from 20 to 35 °C. The intersections between the two curves for that range of temperatures are shown by the bold line, which contains all possible (*n*, *λ*, *T*)m for that calibration set. Figure 1b is a representation of (*n*. *λ*, *T*)_m_ in *λ*, *T* space. The “true” *λ*_m_ at each *T* is given by the solid line in Fig. 1b, with our measurements of *λ*_m_ given by the squares. The *λ*_m_ measurement errors (Δ*λ*_m_), calculated as $\lambda_{m}^{\text{meas}}-\lambda_{m}^{\text{true}}$, are shown by the squares in Fig. 1c. A linear fit to the measurement errors is shown by the solid line in Fig. 1c, and will be discussed later in this paper.

**Fig. 2:**
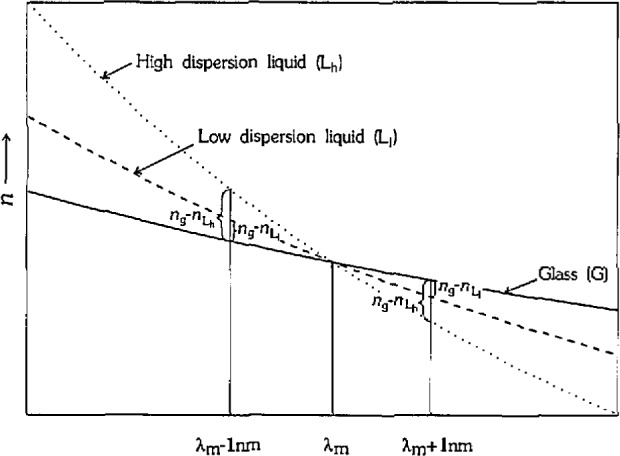
Effect of liquid dispersion on refractive index errors.

The relationship between the error in the measurement of *λ*_m_ and errors in the calculated *n* is dependent on the difference in dispersion between the liquid and the solid. The relationship stems from the fact that the refractive index of the solid is calculated from the dispersion equation of the liquid. Figure 2 illustrates this point for a glass measured in two liquids of different dispersion. For an error of ±1 nm in the determination of *λ*_m_, the error in the calculated *n* is larger when the glass is measured in the high dispersion liquid. This relationship between the error in *n* and the difference in dispersion between the glass and the liquid is important to the discussion of the technique, and will be referred to frequently. We define the difference in dispersion between the glass and liquid as the difference in refractive index between the glass and the liquid at ±1 nm of *λ*_m_ [(*n*_gls_−*n*_liq_) @*λ*_m_ ±lnm]. This value varies slightly with wavelength for each glass/liquid calibration set and is a quantitative description of the relief of the glass grain at ± 1 nm of match conditions.

Other variables in the experiment in addition to *λ,n,T*, and dispersion include the operator and the filter. As mentioned before, the seven calibration sets, comprising seven immersion liquids and four glasses, were chosen for the analysis of the asbestos reference materials, but also allow us to test the effects of differences in dispersion. Three operators performed the measurements, and differences due to operator bias are discussed. The data for the seven calibration sets were collected using the narrow-bandpass GIF with a 2 mm exit slit. Additional measurements were performed using the fixed-wavelength filters, the narrow-bandpass GIF with the slit fully opened, and the broad-bandpass GIF to test the effects of bandpass.

Each operator measured at least two grains of each glass in each liquid from independent preparations. The measurements of *λ*_m_ were performed for each glass grain at three temperatures; one at room temperature, a second between room temperature and 35 °C, and a third at approximately 35 *°*C. The measurements were always made in order of increasing temperature, and three measurements were made at each temperature. The measurements were made when the temperature in the immersion cell had stabilized; the temperature did not vary by more than 0.1 °C for any set of three measurements.

## 4. Results

The (*T*, λ_m_) data collected for each glass/liquid calibration set listed in Table 1 were converted to (Δ*λ*_m_, *T*), as shown in Figs. 1b and 1c, to provide common ground on which to compare all calibration sets. Both the precision and the accuracy of the measurements were determined using the data in this form. The accuracy was additionally determined by converting the (*T, λ*_m_) data to (*λ*, *n*) to determine errors in the dispersion curves calculated from the measured data.

### 4.1 Precision

The precision of the *λ*_m_ measurement, as indicated by the variation of the measurements at each Tin Figs. 1b and 1c, was calculated as one standard deviation (l*σ*) of the mean of the residuals from a linear fit to (Δ*λ*_m_, *T*) for each calibration set. The precision was calculated separately for each operator’s data, and also for the combined set of data. The precision of each operator, and of the combined dataset (all), is given in Table 2, where the glass/liquid calibration sets are identified by *n_d_* of the liquid used. The difference in dispersion between liquid and glass is given by (*n*_gls_ −*n*_liq_) @*λ*_m_ ± 1 nm, which describes quantitatively the relief of the glass in the liquid. The slopes of the two curves change with wavelength, and therefore (*n*_gls_ −*n*_liq_) @*λ*_m_ ± 1 nm varies with wavelength. The range given for (*n*_gls_ −*n*_liq_) @*λ*_m_ ± 1 nm for each calibration set represents a dependence on wavelength, and decreases with increasing wavelength. The range in *λ*_m_ for each calibration set is given in Table 1.

The precision can be interpreted with respect to Fig. 2, in which the solid is measured in either a high dispersion liquid (*V*≥ 0.050) or a low dispersion liquid (*V* = 0.031). (The dispersion of the glass remains relatively constant (*V* = 0.017–0.020), except for calibration set 1.678 which has a higher dispersion glass (*V* = 0.033).) The *λ*_m_ measurements are more precise in the high dispersion liquids than in the low dispersion liquids. The precision is operator dependent, but this basic categorization holds for all three. Operators 1 and 3 have an average l*σ* of approximately 1.5 nm for the high dispersion liquids and an average 1*σ* of approximately 3 nm for the low dispersion liquids. Operator 2 is less precise but follows the same trends. The improvement in precision with increasing liquid dispersion and therefore increasing (*n*_gls_ −*n*_liq_)@*λ*_m_ ± 1 nm indicates that the amount of relief influences the precision of the measurement, which is a reasonable conclusion. The precision also seems to have a lower boundary at l*σ* equal to approximately 1 nm, beyond which the relief can increase without an additional improvement in precision. The precision for set 1.694 is comparable to the other high dispersion liquid sets, even though this set has the highest relief. This probably represents a limitation of the GIF in that it may not be capable of providing peak wavelengths with a separation of better than approximately 1 nm.

The precision in *n* for the measurements can be calculated by multiplying the *λ*_m_ precision by (*n*_gls_ −*n*_liq_) @*λ*_m_ ± 1 nm, which defines the error in *n* for each 1 nm error in *λ*_m_,. The precision in *n*, calculated using the mean value of (*n*_gls_ −*n*_liq_) @*λ*_m_ ± 1 nm for each calibration set, is given in Table 3. The precision in *n* for the low dispersion liquids, where relief is the controlling factor, is between ±1×10^−4^ and ±2.4×10^−4^ for operators 1 and 3. The precision degrades for the high dispersion liquids with the exception of set 1.678, which has a high glass dispersion and thus a lower relief. The precision in *λ*_m_, and thus *n*, for the high relief calibration sets is ultimately controlled by the ~1 nm limitation in Am. Therefore, for calibration set 1.694, the precision in *n* is poor even though the precision in Am is comparable to other sets, due solely to the high relief. Put in another way, to achieve a precision in *n* of approximately ± 1×10^−4^ for calibration set 1.694 requires a precision in the measurement of *λ*_m_ of approximately ± 0.4 nm or better, which appears to be beyond our measurement capabilities.

The precision of the measurements can be summarized as follows: at best we can discriminate changes in relife of approximately 1×10^−4^ and our best precision in the measurement of *λ*_m_ is ~ 1 nm. Because we cannot measure *λ*_m_  with a precision better than ~ 1 nm, a difference in dispersion between the liquid and solid which results in (*n*_gls_ −*n*_liq_) @*λ*_m_ ± 1 nm of greater than ±1×10^−4^, will result in an imprecision in *n* that is larger than ±1×10^−4^

The precision of the *λ*_m_ measurements, as given in Table 2, is described in terms of individual operators, and also in terms of the combined data set. For each calibration set, 1*σ* for the combined dataset is larger than the average of the three operators, indicating a bias among operators. Analysis of the average value of *λ*_m_ at a given temperature for each operator indicates that operator 1 has a consistent negative bias on the order of approximately 1 nm with respect to the other two operators. It is possible to correct this type of problem through retraining of the operator or by the determination of calibration curves for each operator.

### 4.2 Accuracy

**Fig. 3:**
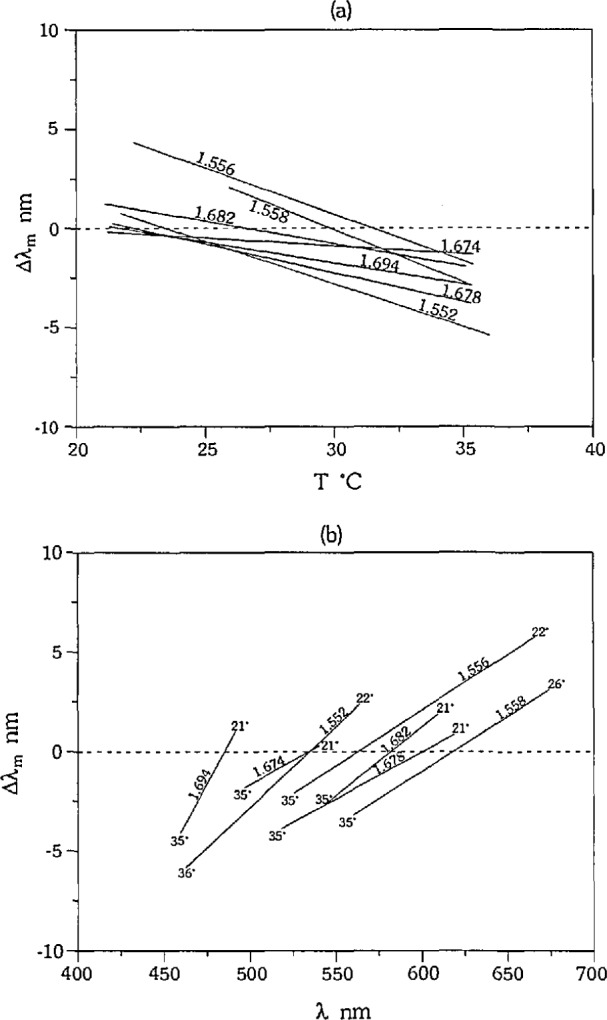
Bias in *λ*_m_: (a) with temperature and (b) with wavelength. Calibration sets identified by *n*_D_ of liquid. Lines represent least squares linear fits to Δ*λ*_m_  for each calibration set.

The accuracy of the technique was analyzed by determining the errors in the measurement of *λ*_m_ with respect to *T* for each glass/liquid calibration set. Systematic errors in the measurements of Am were observed for each calibration set. The linear fits of Δ*λ*_m_ to *T* for each set have negative slopes, and the values of Δ*λ*_m_ predicted by the fits are positive or close to zero at the low temperature end of each set, and negative at the high temperature end of each set, as shown in Fig. 3a. Because of the nature of the experiment, an increase in temperature always corresponds to a decrease in *λ*_m_, and therefore a bias associated with temperature cannot be separated from a bias associated with wavelength. Linear fits to the errors in *λ*_m_ with respect to wavelength for each set are given in Fig. 3b, with temperature listed at each end point. The linear fits all have positive slopes, and the predicted Δ*λ*_m_‘s are negative at the short wavelength end of each set, and positive at the long wavelength end of each set. It is important to note that the calibration sets cover different ranges of wavelength, and that the errors are not associated with wavelength in an absolute sense. These data indicate that the systematic errors are not due to color sensitivity of the detector (human), as we will discuss later in the paper. The linear fits to Δ*λ*_m_ with respect to temperature and wavelength shown in Fig. 3 are noisy, as can be seen in Fig. 1c, and the absolute values of the slope and intercept for each set are not the same. However, the repetition of the trends in the errors for all sets is significant, and indicates that there is a bias in our measurements which correlates with temperature and wavelength.

Some possible sources of error that could produce the observed bias include those related to the heating stage (thermocouple calibration, temperature instability, temperature gradients), systematic errors in the calibration of the GIF, systematic errors in the dispersion of the liquids and/or glasses, and uncertainties in temperature coefficients. The calibrations of the heating stage, GIF, and glasses are described in the calibration section and we can eliminate these factors as possible significant contributors to the observed bias. We were concerned about possible systematic errors in the calibration of the liquids, since we were not able to measure the dispersion independently (other than the measurement of F − C). The dispersions of the liquids also change with temperature such that d*n/*d*T* is not the same at all wavelengths, although by a small amount (10^−5^). In addition, the temperature coefficients of the glasses are not known, although they are also small (10^−6^).

The possibility that systematic errors in the dispersions of the liquids and uncertainties in temperature coefficients were responsible for the bias observed in *λ*_m_ was tested by using a liquid and glass for which those quantities are well characterized. We measured SRM 1822, an optical glass refractive index standard, in SRM 18231, one of the refractive index liquid standards. The dispersions of both liquid and glass are well characterized, as are the temperature coefficients of the liquid with respect to wavelength. SRM 18231 is a silicone oil that is chemically and thermally stable. The refractive index of the liquid was measured using the minimum deviation technique and is certified at 10 wavelengths in the visible with an accuracy of ±4×10^−5^ at four temperatures from 20 to 80°C. SRM 1822 is a commercial soda-lime glass that is certified for refractive index at 13 wavelengths in the visible with an accuracy of ±9 × 10^−6^. The temperature coefficient for SRM 1822 is not given on the certificate, however, the temperature coefficients for common optical glasses are available from the Schott catalog [13] and range from − 3 × l0^−6^ to −5 ×10^−6^, increasing with increasing refractive index. A barium crown glass (BK7) made by Schott Glass is similar in refractive index to SRM 1822, and has a temperature coefficient of −3×l0^−6^. This value was taken as the temperature coefficient for SRM 1822, although the selection of other temperature coefficients within the range listed produced no significant difference in results.

**Fig. 4:**
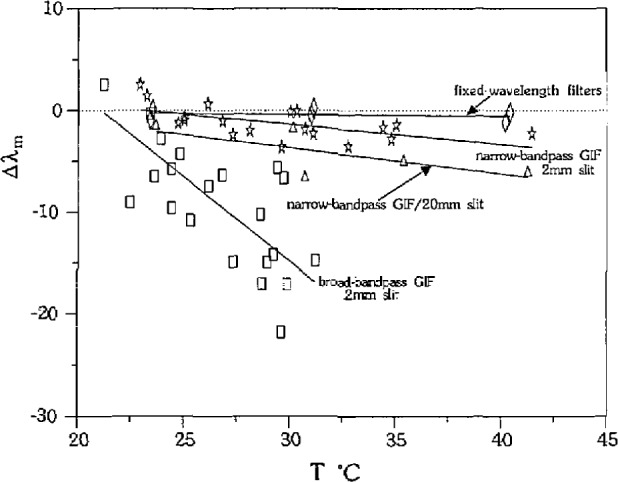
Effects of bandpass on measurement bias for measurements of SRM 1822 in SRM 18231. The broad-bandpass GIF (squares) has a 30 nm FWHM, the narrow-bandpass GIF has a 15 nm FWHM (stars) which increases to 30 nm with the slit fully open (triangles). The fixed-wavelength filters each have a 10 nm FWHM (diamonds).

The errors in the measurements of SRM 1822 in SRM 18231 using the narrow bandpass GIF are shown in Figs. 4, and the linear fit to the errors follows the same trend as the fits to the errors for all other calibration sets. The conclusion from these measurements is that the bias in the measurements of our seven calibration sets, as displayed in Fig. 3, is not due to uncertainties in dispersion, temperature coefficients, or liquid instability at high temperatures.

We were able to change the magnitude of the bias observed in *λ*_m_ by changing the bandpass of the GIF. We performed measurements of SRM 1822 using the narrow bandpass GIF with the slit fully open (FWHM ~30 *nm*), and the broad bandpass GIF, and the errors are illustrated in Fig. 4. The linear fits to the data from the GIFs have negative slopes, and the magnitude of the slope increases with increasing bandpass. The slopes of the three linear fits are significantly different from each other such that at 30 °C the 95% confidence limits for each fit comprise separate populations.

We do not see a bias with temperature or wavelength when we use the fixed wavelength filters (10 nm FWHM) and vary the temperature to measure *T_m_* (single variation technique). Measurements of SRM 1822 using the 546.1, 486.1, and 435.8 nm filters are shown in Fig. 4 [after conversion to (*T*, Δ*λ*_m_)] along with a least squares linear fit. A 95% confidence interval of the slope of the fit includes zero, indicating that there is no significant bias for this data.

Measurements of the other calibration sets using the fixed-wavelength filters also do not exhibit any systematic errors. Due to the spacing of the transmittance peaks of the filters, we are usually limited to one or two temperature measurements for each calibration set, and therefore, we have smaller data sets than for the GIFs. However, the measurements from the fixed-wavelength filters span the full temperature and wavelength range, and we do not observe a bias with respect to temperature or wavelength. This result conflicts with that of Ref. [7] which reports the same refractive index bias for both fixed-wavelength filters and a GIF. The authors do not state the bandpass of their filters, and it is possible that the different results are due to this factor. We did not test any fixed-wavelength filters with a broader bandpass. We did test the possibility that the variation of wavelength was responsible for the bias by fixing the position of the GIF and performing a single variation measurement, as with the fixed-wavelength filters. The data collected with the GIF in a fixed position were biased in the same fashion as the data collected by varying the wavelength.

### 4.3 Dispersion Calculations

Given the fact that there is a bias in the measurement of *λ*_m_, we need to determine the effect of this bias on the calculated refractive indices and fitted dispersion curves. Qualitatively, the errors in *n* always have the opposite sign of Δ*λ*_m_, therefore Δ*n* is positive for the short wavelength–high temperature end of each calibration set, and negative for the long wavelength–low temperature end of each calibration set. We can get a general idea of the magnitude of Δ*n* using the same procedure we used for determining the precision, namely by multiplying the error in *λ*_m_  by (*n*_gls_−*n*_liq_)@*λ*_m_ ± 1 nm for that calibration set. For example, from Fig. 3b we can see that at the short wavelength end of each calibration set, the predicted errors in *λ*_m_ from the linear fits range from approximately −2 nm to −6 nm, and we will assume an average error of − 4 nm for discussion purposes only. If we multiply − 4 nm by the largest value of (*n*_gls_−*n*_liq_)@*λ*_m_ ± 1 nm for each set from Table 2 (which corresponds to the short wavelength end) we see that the errors in *n* will range from +2× 10^−4^ to +1.2× 10^−3^ As discussed with reference to precision, the errors in the calculated *n* increase as the difference in dispersion between the glass and the liquid increase, even for the same error in *λ*_m_. This fundamental association stems from the assumption of the technique that the measured value lies on the liquid dispersion curve.

**Fig. 5:**
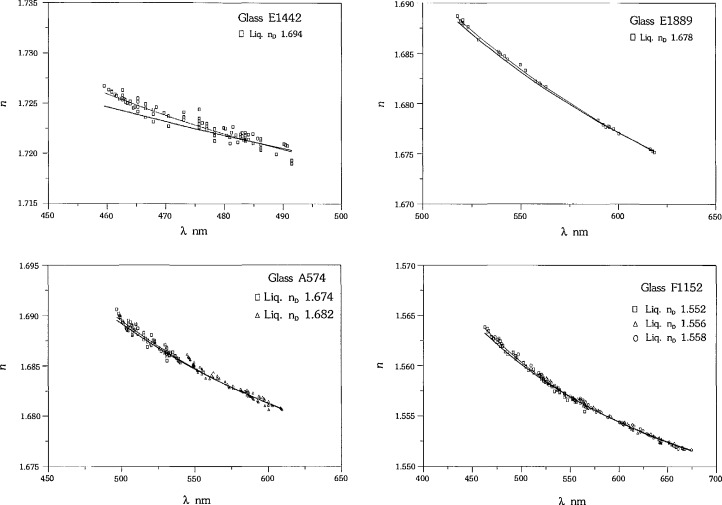
Measurements of calibration glasses with fitted Cauchy (thin line) vs true (bold line).

**Fig. 6:**
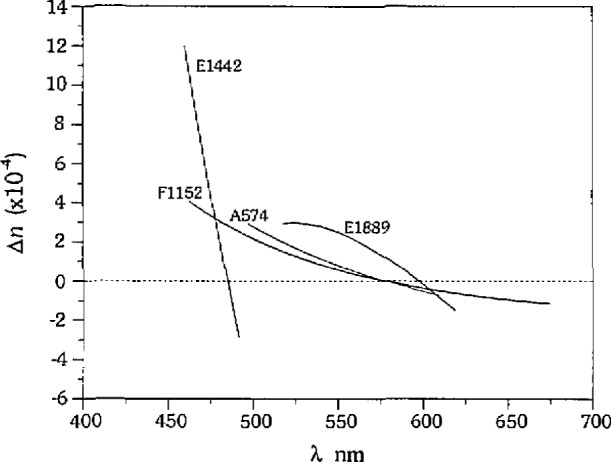
Dispersion curve measurement errors for calibration glasses.

The ultimate goal of the measurement technique is to calculate dispersion constants for the unknown to predict the refractive index throughout the measurement range. We must, therefore, determine the errors involved in the calculation of the dispersion of the calibration glasses. The dispersion of each glass listed in Table 1 was calculated by converting the measurements from each liquid to (*λ*,*n*), combining the data from each liquid, and fitting the combined set to a Cauchy equation. In the case of glass F1152 there are three sets of data which are combined for the fit, for glass A574 there are two sets of data, and for glasses E1889 and E1442 there is only one set of data each. The (*λ*,*n*) fits to the data for each glass are shown in Fig. 5, along with the “true” (*λ*,*n*) shown by the bold line. The error in *n* at each A is determined by subtracting the “true” *n* at each wavelength from the *n* predicted by the fit to the data. The Δ*n*’s thus calculated for the glasses are shown in Fig. 6. This is the same approach recommended by Su et al. (1987), who use three liquids to measure each calibration glass.

**Fig. 7:**
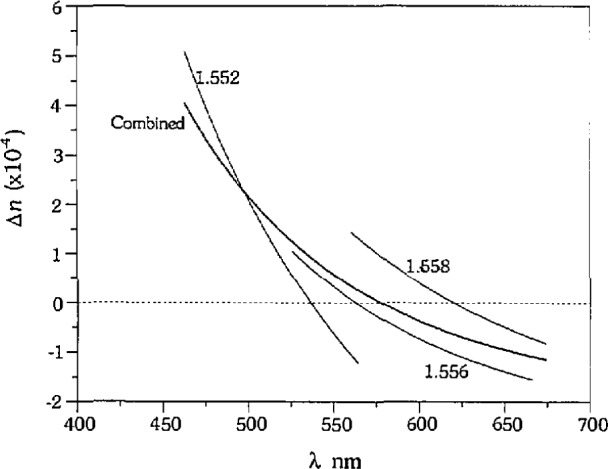
Dispersion curve errors from multiple vs single liquids. Dispersion curve measurement errors for glass F1152 determined for each liquid (thin lines) and for combined dataset (bold line).

The errors in refractive index calculated using this approach now exhibit the systematic bias discussed by Refs. [6–8], particularly for glasses F1152 and A574, which are biased high for short wavelengths and biased low for long wavelengths, approaching zero error at approximately 550 nm. As discussed earlier, however, the bias does not correlate with absolute wavelength, but with the minimum and maximum wavelength of each glass/liquid cahbration set. The apparent correlation of the bias with absolute wavelength for glasses F1152 and A574 results from the combination of the data from multiple liquids, and the overlap of the sets. If the data from each calibration set for glass Fl 152 are treated separately, we obtain the Δ*n_λ_* curves shown by the thin lines in Fig. 7, whereas if we combine the sets we obtain the Δ*n_λ_* curve shown by the bold line in Fig. 7. The error in refractive index at a particular wavelength is dependent upon the number of liquids used to measure the glass; the bias in each set of data from a given liquid will be compensated over the wavelength range where there is overlap with data from another liquid.

The dependence of Δ*n_λ_* on the number of liquids used in the calibration measurements and on the difference in dispersion between the liquid and the solid indicates that the use of such values to correct measurements of unknowns is only appropriate when the unknown has the same dispersion as the calibration material (or the difference in dispersion between liquid and solid is the same for both) and when the same number of liquids covering the same general range of wavelengths are used. For example, if only one liquid were used to determine Δ*n*_560_ for glass F1152, the result would be either −1 ×10^−4^, 0, or +1.5 ×10^−4^, depending on the liquid used for calibration. In addition, glass E1889 would be an inappropriate calibration material to use to correct the measurements of glass A574 even though they are similar in refractive index, due to the large difference in dispersion.

**Fig. 8:**
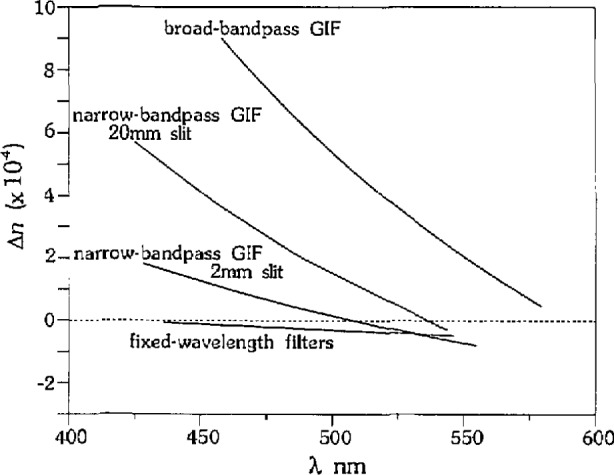
Effect of bandpass on refractive index errors using data displayed in Fig. 4.

The refractive index errors are also highly dependent on the filter used because of the bias associated with bandpass. Figure 8 shows the calculated errors in *n* for glass SRM 1822, using the data from Fig. 4. The error in refractive index for the fixed wavelength filters and the narrow bandpass GIF with the 2 mm slit is within ±2×10^−4^, but increases to approximately +1 × 10^−3^ for the broad bandpass GIF.

## 5. Discussion

We believe that the bias we observe in *λ*_m_ may explain the bias observed in refractive index in the previous studies [6–8]. This bias correlates with temperature and wavelength, but cannot be traced to calibration or measurement errors associated with either variable. The magnitude of the bias appears to be directly influenced by the bandpass of the interference filter. We do not observe a bias with the 10 nm FWHM fbced wavelength filters, but we observe a bias with the variable wavelength GIFs that increases with increasing bandpass. We did not test fixed wavelength filters with wider bandpasses and the question remains as to whether the GIFs themselves are a source of the bias, with the bandpass of the GIFs as an additional factor, or whether it is bandpass alone (or another variable associated with the transmission of the filter) which is the critical factor. The GIFs and the fixed wavelength filters are similar in that both are interference filters, but are dissimilar in transmission characteristics, due to the gradation in thickness of the GIF. Louisnathan et al. (1978) report a bias using fixed wavelength filters of unspecified bandpass, and it is therefore probable that the bias is not restricted to the use of GIFs. We are perplexed by the apparent association between bandpass and the observed bias, and can only suggest that the relief of the glass grains must be affected by the bandpass in some manner that biases our selection of *λ*_m_.

We found that even with the bias in the technique, the measurements of the glasses were acceptable (within ±5 ×10^−4^) with the exception of glass E1442, for which we have a maximum error of + 1.2×10^−3^ and a precision of ±6 ×10^−4^. The large error in refractive index is due to the large difference in dispersion between the glass and the liquid, and not to any degradation in the actual measurements. This calibration set emphasizes the need to select liquids with the lowest dispersion possible although the choices are usually quite limited. In the initial stages of this project, when we were determining accuracy and precision in the conventional manner by analyzing the errors in refractive index with wavelength, we mistakenly thought that the precision and accuracy of the technique were dependent on the absolute refractive index of the glass as our errors increased with increasing refractive index. Only after we adopted the approach of assessing the errors in *λ*_m_ did we realize that the correlation was with the difference in dispersion between the liquid and the glass, which generally increases with increasing refractive index.

Because of the general acceptability of the double variation measurements, we felt it was appropriate to use the technique for the measurement of the asbestos reference materials. We could have used the fixed-wavelength filters and the single variation technique, for which we did not detect a bias, but our heating stage is not designed for rapid oscillation and equilibration of temperature which is necessary for efficient single variation measurements. We do not correct the measurements of our unknowns using the calibration measurements because of the strong dependence of Δ*n_λ_* on the specifics of each calibration set. We do use the calibration measurements to provide estimates of the errors in our measurements and to determine whether our variables are under control during measurement of the unknowns.

## 6. Conclusions

The errors in refractive index measurement using the double variation technique were determined by characterizing the accuracy and precision of measuring the matching wavelength (*λ*_m_). The precision and accuracy of the technique in terms of the measurement of refractive index are ultimately dependent on the difference in dispersion between the solid and the liquid. The best precision possible, which in our case is 1 or 2 ×10^−4^, is dependent on the operator’s ability to perceive changes in relief, and is only possible for those liquid/solid combinations where (*n*_gls_−*n*_liq_)@*λ*_m_ + l nm < 1 ×10^−4^. This limitation is based on our inability to measure *λ*_m_ with a precision better than approximately ± 1 nm, which may represent a limitation of the filter.

There is a bias in our measurements of Am using a GIF which correlates with temperature and wavelength. The bias results in positive refractive index errors at the short wavelength–high temperature end of each dataset, and negative refractive index errors at the long wavelength—low temperature end of each dataset. (A dataset is defined as the measurements of 1 glass in 1 liquid). The bias does not correlate with absolute wavelength, and therefore does not appear to result from a relative sensitivity of the human eye. The magnitude of the bias increases with increasing bandpass, as determined by opening the exit slit of the narrow-bandpass GIF, and by using a GIF with a broader bandpass. The bias, in association with a glass and liquid with a large difference in dispersion, can result in errors in refractive index that exceed ±5×10^−4^.

In general, our errors in refractive index using the narrow-bandpass GIF (15 nm FWHM) are within ±5×10^−4^throughout the visible spectrum, and within ± 1 ×10^−4^ at 589.3 nm, as calculated from fits to the combined data from multiple liquids. Correction factors calculated from the measurements of calibration glasses are dependent on the number of liquids used and the dispersion of both liquid and solid, and should ideally be used only when these characteristics are matched in the measurement of the unknown.

## Figures and Tables

**Table 1:** Calibration sets

Liquid *n*_D_	Glass	Glass *n*_D_	Glass *V*^a^	*λ*_m_ Range(nm)
1.552	F1152	1.5549	0.017	463–564
1.556	F1152	1.5549	0.017	526–666
1.558	F1152	1.5549	0.017	560–674
1.674	A574	1.6820	0.018	497–540
1.678	E1889	1.6783	0.033	518–619
1.682	A574	1.6820	0.018	545–609
1.694	E1442	1.7110	0.020	460–491

a Dispersive power *V=n*_F_*−n*_C_*/n*_D_*−*1 (Jenkins and White, 1976).

**Table 2:** *λ*_m_ precision

Set	Op1	Op2 1*σλ*_m_ (nm)	Op3	All^a^	Liq*V*^b^	(*n*_gls_ −*n*_liq_)@*λ*_m_ ± 1 nm×10^−4^
1.552	±2.8	±3.7	±2.2	±3.3	0.031	0.9−0.8
1.556	±2.8	±3.5	±3.1	±4.6	0.031	0.6−0.3
1.558	±3.1	±3.5	±3.2	±3.5	0.031	0.5−0.3
1.674	±1.7	±2.0	±1.3	±2.0	0.050	1.9−1.7
1.678	±1.5	±2.9	±1.1	±2.0	0.050	0.9−0.6
1.682	±1.6	±2.7	±1.7	±2.3	0.053	1.4−1.0
1.694	±1.6	±2.9	±1.3	±2.4	0.053	3.1−2.3

a Determined from combined dataset.

b Dispersive power *V=n*_F_*−n*_C_*/n*_D_*−*1.

**Table 3:** *n* measurement precision

Set	Op 1	Op 2 1*σn* (×10^−4^)	Op 3	All
1.552	±2.4	±3.1	±1.9	±2.8
1.556	±1.3	±1.6	±1.4	±2.1
1.558	±1.2	±1.4	±1.3	±1.4
1.674	±3.1	±3.6	±2.3	±3.6
1.678	±1.1	±2.2	±0.8	±1.2
1.682	±1.9	±3.2	±2.0	±2.8
1.694	±4.3	±7.8	±3.5	±6.5

## Footnotes

1 Certain commercial equipment, instruments, or materials are identified in this paper to specify adequately the experimental procedure. Such identification does not imply recommendation or endorsement by the National Institute of Standards and Technology, nor does it imply that the materials or equipment identified are necessarily the best available for the purpose.
